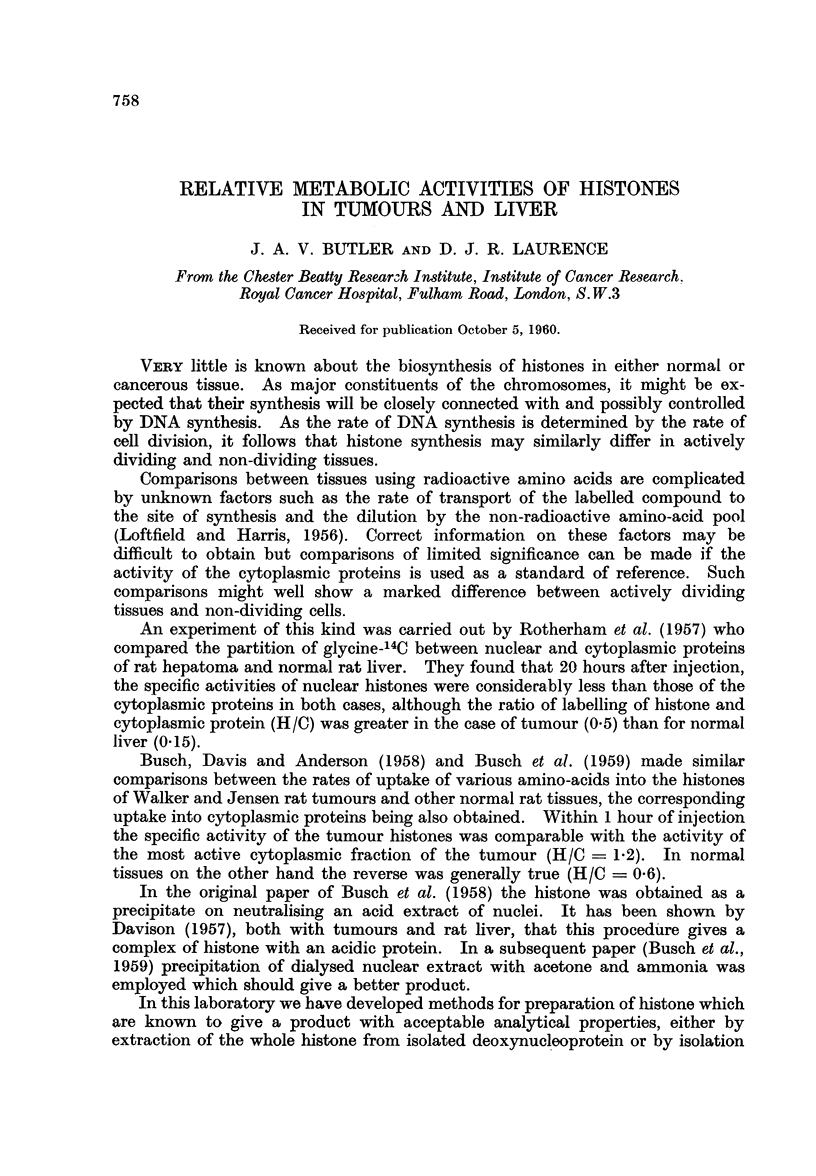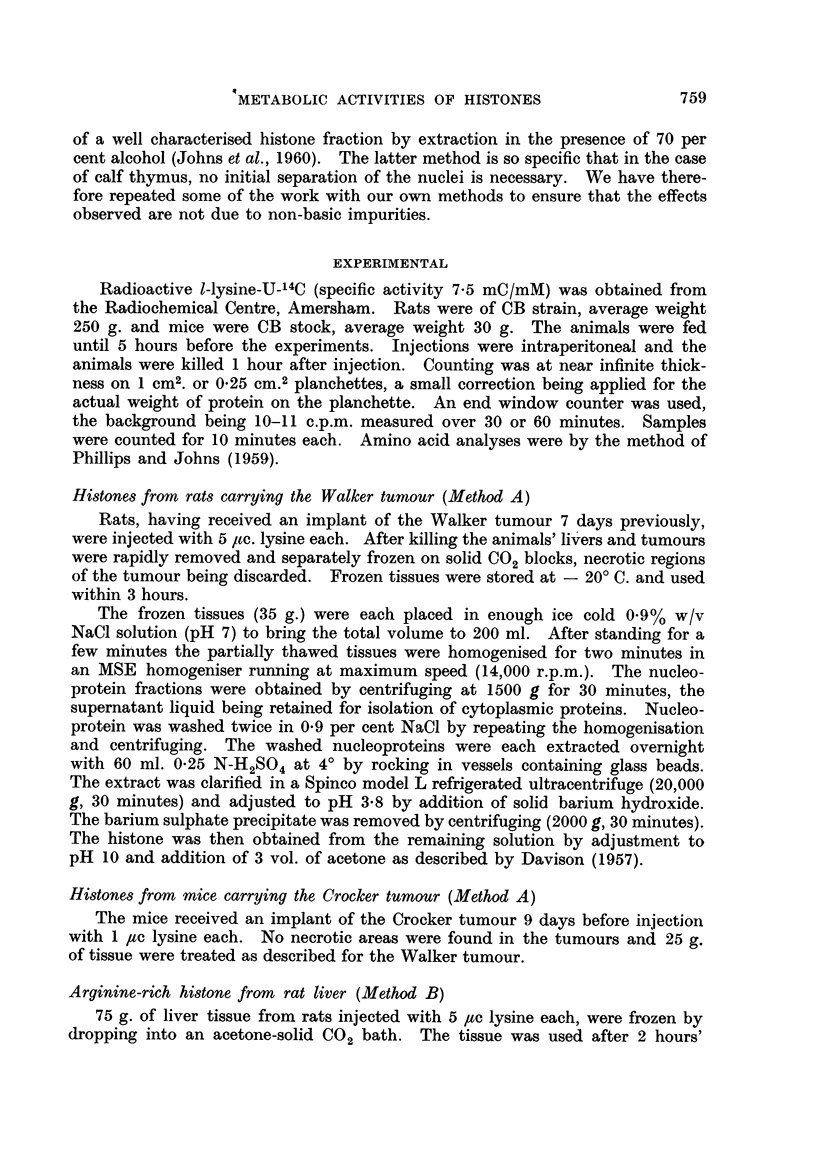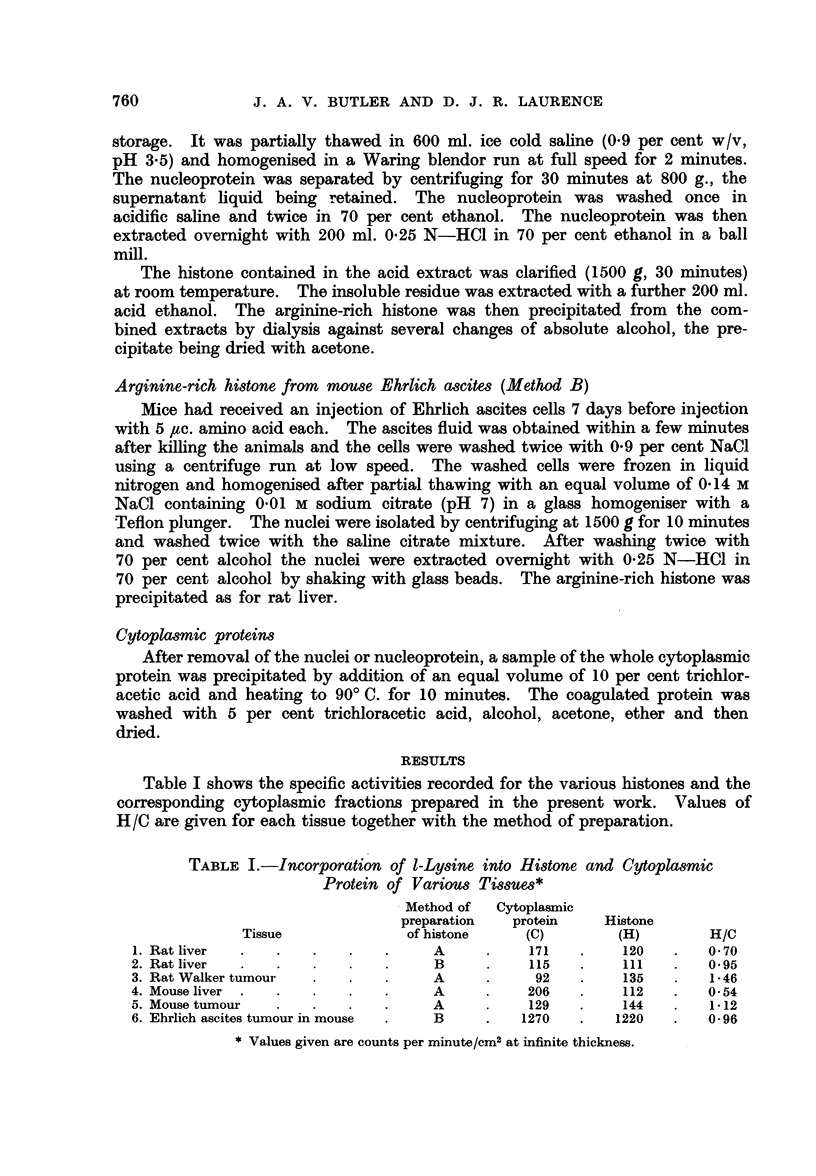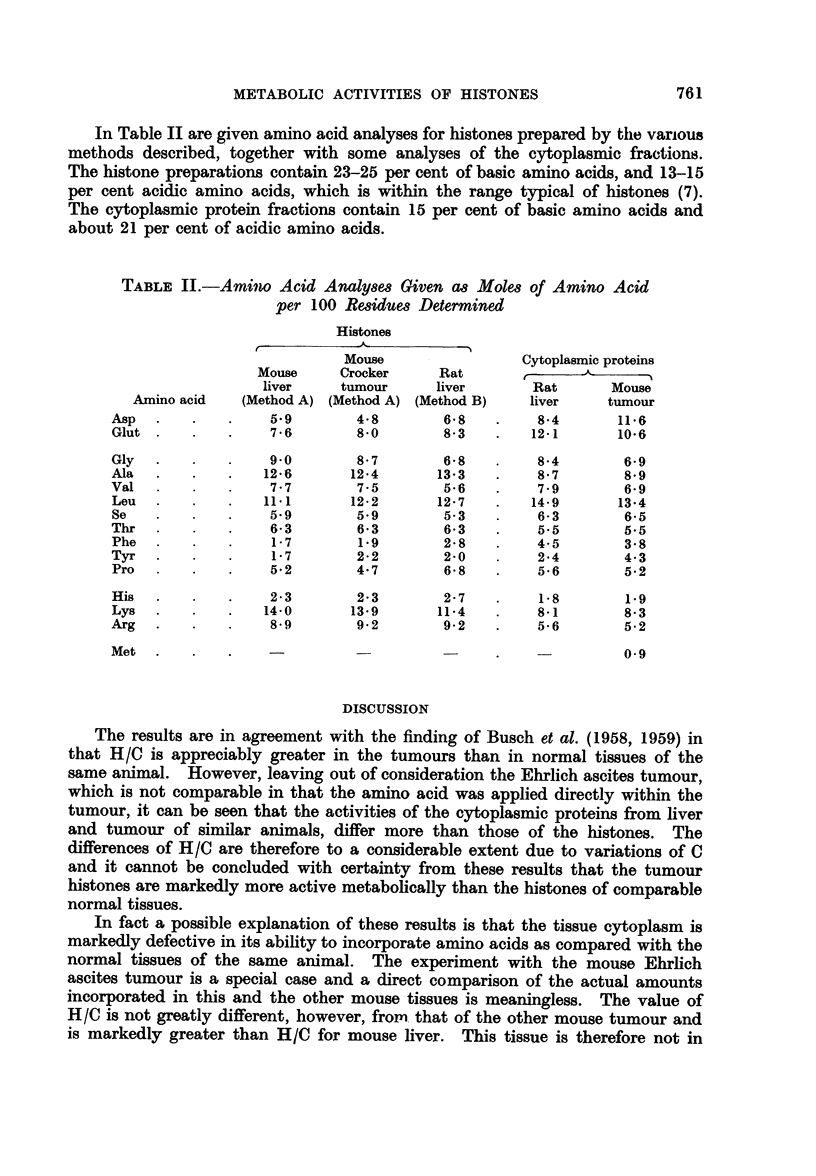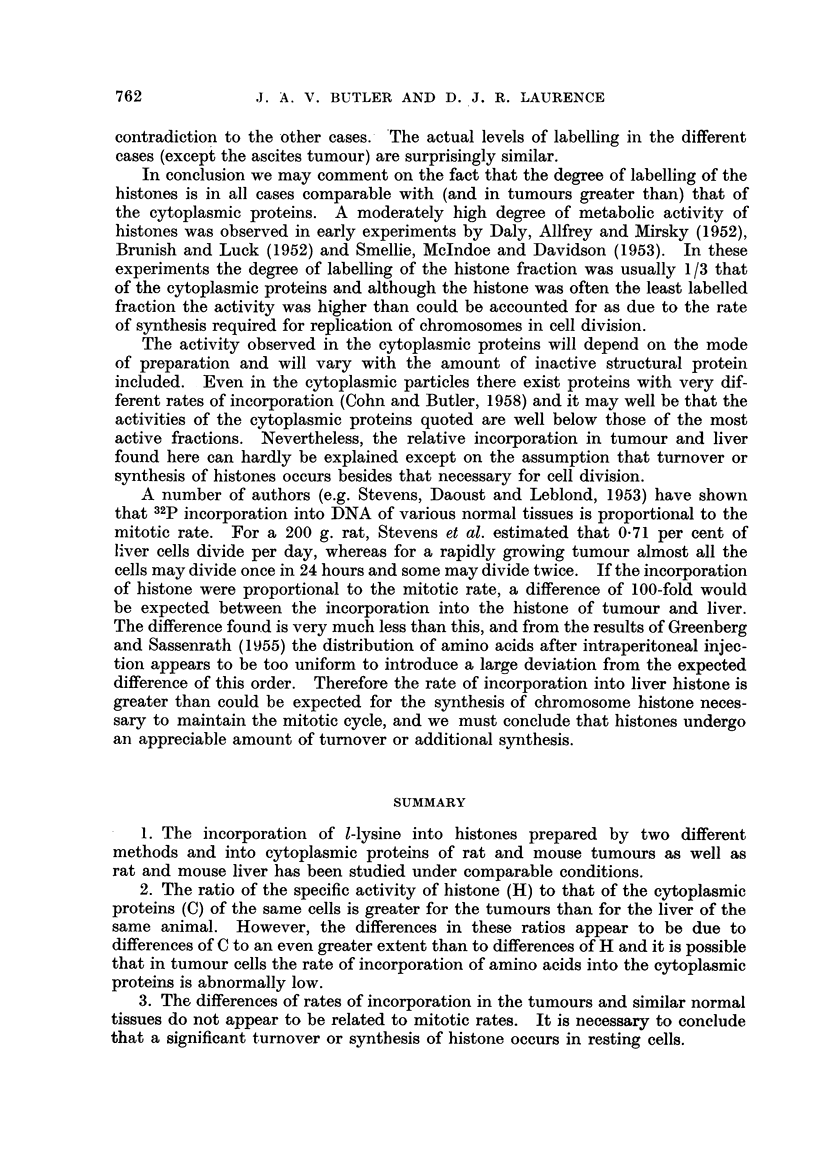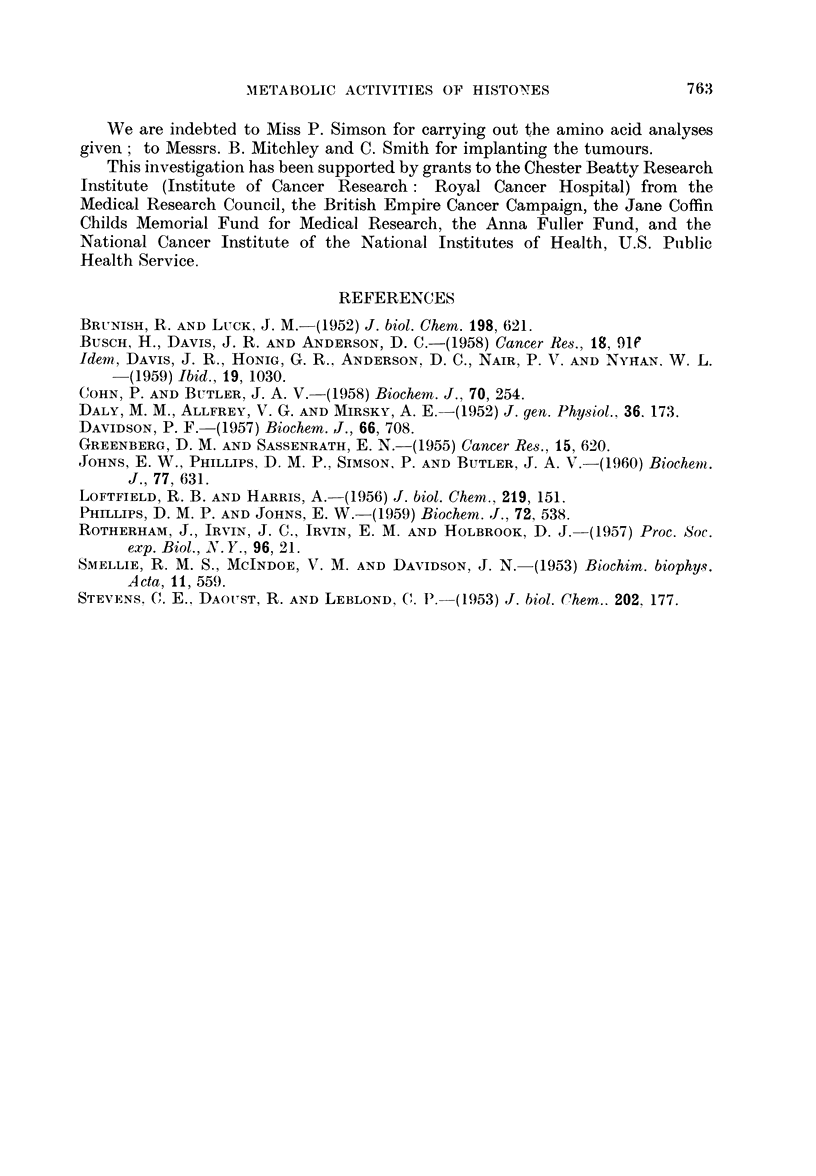# Relative Metabolic Activities of Histones in Tumours and Liver

**DOI:** 10.1038/bjc.1960.86

**Published:** 1960-12

**Authors:** J. A. V. Butler, D. J. R. Laurence


### RELATIVE METABOLIC ACTIVITIES OF HISTONES IN TUMOURS AND LIVER

J. A. V. BUTLER AND D. J. R. LAURENCE

*From the Chester Beatty Research Institute, Institute of Cancer Research, Royal Cancer Hospital, Fulham Road, London, S.W.3*

Received for publication October 5, 1960.

VERY little is known about the biosynthesis of histones in either normal or cancerous tissue. As major constituents of the chromosomes, it might be expected that their synthesis will be closely connected with and possibly controlled by DNA synthesis. As the rate of DNA synthesis is determined by the rate of cell division, it follows that histone synthesis may similarly differ in actively dividing and non-dividing tissues.

Comparisons between tissues using radioactive amino acids are complicated by unknown factors such as the rate of transport of the labelled compound to the site of synthesis and the dilution by the non-radioactive amino-acid pool (Loftfield and Harris, 1956). Correct information on these factors may be difficult to obtain but comparisons of limited significance can be made if the activity of the cytoplasmic proteins is used as a standard of reference. Such comparisons might well show a marked difference between actively dividing tissues and non-dividing cells.

An experiment of this kind was carried out by Rotherham *et al.* (1957) who compared the partition of glycine-$^{14}$C between nuclear and cytoplasmic proteins of rat hepatoma and normal rat liver. They found that 20 hours after injection, the specific activities of nuclear histones were considerably less than those of the cytoplasmic proteins in both cases, although the ratio of labelling of histone and cytoplasmic protein (H/C) was greater in the case of tumour (0·5) than for normal liver (0·15).

Busch, Davis and Anderson (1958) and Busch *et al.* (1959) made similar comparisons between the rates of uptake of various amino-acids into the histones of Walker and Jensen rat tumours and other normal rat tissues, the corresponding uptake into cytoplasmic proteins being also obtained. Within 1 hour of injection the specific activity of the tumour histones was comparable with the activity of the most active cytoplasmic fraction of the tumour (H/C = 1·2). In normal tissues on the other hand the reverse was generally true (H/C = 0·6).

In the original paper of Busch *et al.* (1958) the histone was obtained as a precipitate on neutralising an acid extract of nuclei. It has been shown by Davison (1957), both with tumours and rat liver, that this procedure gives a complex of histone with an acidic protein. In a subsequent paper (Busch *et al.*, 1959) precipitation of dialysed nuclear extract with acetone and ammonia was employed which should give a better product.

In this laboratory we have developed methods for preparation of histone which are known to give a product with acceptable analytical properties, either by extraction of the whole histone from isolated deoxynucleoprotein or by isolation of a well characterised histone fraction by extraction in the presence of 70 per cent alcohol (Johns *et al.*, 1960). The latter method is so specific that in the case of calf thymus, no initial separation of the nuclei is necessary. We have therefore repeated some of the work with our own methods to ensure that the effects observed are not due to non-basic impurities.

### EXPERIMENTAL

Radioactive *l*-lysine-U-$^{14}$C (specific activity 7·5 mC/mM) was obtained from the Radiochemical Centre, Amersham. Rats were of CB strain, average weight 250 g. and mice were CB stock, average weight 30 g. The animals were fed until 5 hours before the experiments. Injections were intraperitoneal and the animals were killed 1 hour after injection. Counting was at near infinite thickness on 1 cm$^2$. or 0·25 cm.$^2$ planchettes, a small correction being applied for the actual weight of protein on the planchette. An end window counter was used, the background being 10–11 c.p.m. measured over 30 or 60 minutes. Samples were counted for 10 minutes each. Amino acid analyses were by the method of Phillips and Johns (1959).

### *Histones from rats carrying the Walker tumour (Method A)*

Rats, having received an implant of the Walker tumour 7 days previously, were injected with 5 $\mu$c. lysine each. After killing the animals' livers and tumours were rapidly removed and separately frozen on solid $CO_2$ blocks, necrotic regions of the tumour being discarded. Frozen tissues were stored at $-$ 20° C. and used within 3 hours.

The frozen tissues (35 g.) were each placed in enough ice cold 0·9% w/v NaCl solution (pH 7) to bring the total volume to 200 ml. After standing for a few minutes the partially thawed tissues were homogenised for two minutes in an MSE homogeniser running at maximum speed (14,000 r.p.m.). The nucleoprotein fractions were obtained by centrifuging at 1500 *g* for 30 minutes, the supernatant liquid being retained for isolation of cytoplasmic proteins. Nucleoprotein was washed twice in 0·9 per cent NaCl by repeating the homogenisation and centrifuging. The washed nucleoproteins were each extracted overnight with 60 ml. 0·25 N-$H_2SO_4$ at 4° by rocking in vessels containing glass beads. The extract was clarified in a Spinco model L refrigerated ultracentrifuge (20,000 *g*, 30 minutes) and adjusted to pH 3·8 by addition of solid barium hydroxide. The barium sulphate precipitate was removed by centrifuging (2000 *g*, 30 minutes). The histone was then obtained from the remaining solution by adjustment to pH 10 and addition of 3 vol. of acetone as described by Davison (1957).

### *Histones from mice carrying the Crocker tumour (Method A)*

The mice received an implant of the Crocker tumour 9 days before injection with 1 $\mu$c lysine each. No necrotic areas were found in the tumours and 25 g. of tissue were treated as described for the Walker tumour.

### *Arginine-rich histone from rat liver (Method B)*

75 g. of liver tissue from rats injected with 5 $\mu$c lysine each, were frozen by dropping into an acetone-solid $CO_2$ bath. The tissue was used after 2 hours'

storage. It was partially thawed in 600 ml. ice cold saline (0·9 per cent w/v, pH 3·5) and homogenised in a Waring blendor run at full speed for 2 minutes. The nucleoprotein was separated by centrifuging for 30 minutes at 800 g., the supernatant liquid being retained. The nucleoprotein was washed once in acidific saline and twice in 70 per cent ethanol. The nucleoprotein was then extracted overnight with 200 ml. 0·25 N—HCl in 70 per cent ethanol in a ball mill.

The histone contained in the acid extract was clarified (1500 *g*, 30 minutes) at room temperature. The insoluble residue was extracted with a further 200 ml. acid ethanol. The arginine-rich histone was then precipitated from the combined extracts by dialysis against several changes of absolute alcohol, the precipitate being dried with acetone.

*Arginine-rich histone from mouse Ehrlich ascites (Method B)*

Mice had received an injection of Ehrlich ascites cells 7 days before injection with 5 $\mu$c. amino acid each. The ascites fluid was obtained within a few minutes after killing the animals and the cells were washed twice with 0·9 per cent NaCl using a centrifuge run at low speed. The washed cells were frozen in liquid nitrogen and homogenised after partial thawing with an equal volume of 0·14 M NaCl containing 0·01 M sodium citrate (pH 7) in a glass homogeniser with a Teflon plunger. The nuclei were isolated by centrifuging at 1500 *g* for 10 minutes and washed twice with the saline citrate mixture. After washing twice with 70 per cent alcohol the nuclei were extracted overnight with 0·25 N—HCl in 70 per cent alcohol by shaking with glass beads. The arginine-rich histone was precipitated as for rat liver.

*Cytoplasmic proteins*

After removal of the nuclei or nucleoprotein, a sample of the whole cytoplasmic protein was precipitated by addition of an equal volume of 10 per cent trichloracetic acid and heating to 90° C. for 10 minutes. The coagulated protein was washed with 5 per cent trichloracetic acid, alcohol, acetone, ether and then dried.

### RESULTS

Table I shows the specific activities recorded for the various histones and the corresponding cytoplasmic fractions prepared in the present work. Values of H/C are given for each tissue together with the method of preparation.

TABLE I.—*Incorporation of l-Lysine into Histone and Cytoplasmic Protein of Various Tissues**

| Tissue | Method of preparation of histone | Cytoplasmic protein (C) | Histone (H) | H/C |
|---|---|---|---|---|
| 1. Rat liver | A | 171 | 120 | 0·70 |
| 2. Rat liver | B | 115 | 111 | 0·95 |
| 3. Rat Walker tumour | A | 92 | 135 | 1·46 |
| 4. Mouse liver | A | 206 | 112 | 0·54 |
| 5. Mouse tumour | A | 129 | 144 | 1·12 |
| 6. Ehrlich ascites tumour in mouse | B | 1270 | 1220 | 0·96 |

* Values given are counts per minute/cm$^2$ at infinite thickness.

In Table II are given amino acid analyses for histones prepared by the various methods described, together with some analyses of the cytoplasmic fractions. The histone preparations contain 23–25 per cent of basic amino acids, and 13–15 per cent acidic amino acids, which is within the range typical of histones (7). The cytoplasmic protein fractions contain 15 per cent of basic amino acids and about 21 per cent of acidic amino acids.

TABLE II.—*Amino Acid Analyses Given as Moles of Amino Acid per* 100 *Residues Determined*

| | Histones | | | Cytoplasmic proteins | |
|---|---|---|---|---|---|
| Amino acid | Mouse liver (Method A) | Mouse Crocker tumour (Method A) | Rat liver (Method B) | Rat liver | Mouse tumour |
| Asp | 5·9 | 4·8 | 6·8 | 8·4 | 11·6 |
| Glut | 7·6 | 8·0 | 8·3 | 12·1 | 10·6 |
| Gly | 9·0 | 8·7 | 6·8 | 8·4 | 6·9 |
| Ala | 12·6 | 12·4 | 13·3 | 8·7 | 8·9 |
| Val | 7·7 | 7·5 | 5·6 | 7·9 | 6·9 |
| Leu | 11·1 | 12·2 | 12·7 | 14·9 | 13·4 |
| Se | 5·9 | 5·9 | 5·3 | 6·3 | 6·5 |
| Thr | 6·3 | 6·3 | 6·3 | 5·5 | 5·5 |
| Phe | 1·7 | 1·9 | 2·8 | 4·5 | 3·8 |
| Tyr | 1·7 | 2·2 | 2·0 | 2·4 | 4·3 |
| Pro | 5·2 | 4·7 | 6·8 | 5·6 | 5·2 |
| His | 2·3 | 2·3 | 2·7 | 1·8 | 1·9 |
| Lys | 14·0 | 13·9 | 11·4 | 8·1 | 8·3 |
| Arg | 8·9 | 9·2 | 9·2 | 5·6 | 5·2 |
| Met | — | — | — | — | 0·9 |

### DISCUSSION

The results are in agreement with the finding of Busch *et al.* (1958, 1959) in that H/C is appreciably greater in the tumours than in normal tissues of the same animal. However, leaving out of consideration the Ehrlich ascites tumour, which is not comparable in that the amino acid was applied directly within the tumour, it can be seen that the activities of the cytoplasmic proteins from liver and tumour of similar animals, differ more than those of the histones. The differences of H/C are therefore to a considerable extent due to variations of C and it cannot be concluded with certainty from these results that the tumour histones are markedly more active metabolically than the histones of comparable normal tissues.

In fact a possible explanation of these results is that the tissue cytoplasm is markedly defective in its ability to incorporate amino acids as compared with the normal tissues of the same animal. The experiment with the mouse Ehrlich ascites tumour is a special case and a direct comparison of the actual amounts incorporated in this and the other mouse tissues is meaningless. The value of H/C is not greatly different, however, from that of the other mouse tumour and is markedly greater than H/C for mouse liver. This tissue is therefore not in contradiction to the other cases. The actual levels of labelling in the different cases (except the ascites tumour) are surprisingly similar.

In conclusion we may comment on the fact that the degree of labelling of the histones is in all cases comparable with (and in tumours greater than) that of the cytoplasmic proteins. A moderately high degree of metabolic activity of histones was observed in early experiments by Daly, Allfrey and Mirsky (1952), Brunish and Luck (1952) and Smellie, McIndoe and Davidson (1953). In these experiments the degree of labelling of the histone fraction was usually 1/3 that of the cytoplasmic proteins and although the histone was often the least labelled fraction the activity was higher than could be accounted for as due to the rate of synthesis required for replication of chromosomes in cell division.

The activity observed in the cytoplasmic proteins will depend on the mode of preparation and will vary with the amount of inactive structural protein included. Even in the cytoplasmic particles there exist proteins with very different rates of incorporation (Cohn and Butler, 1958) and it may well be that the activities of the cytoplasmic proteins quoted are well below those of the most active fractions. Nevertheless, the relative incorporation in tumour and liver found here can hardly be explained except on the assumption that turnover or synthesis of histones occurs besides that necessary for cell division.

A number of authors (e.g. Stevens, Daoust and Leblond, 1953) have shown that $^{32}P$ incorporation into DNA of various normal tissues is proportional to the mitotic rate. For a 200 g. rat, Stevens *et al.* estimated that 0·71 per cent of liver cells divide per day, whereas for a rapidly growing tumour almost all the cells may divide once in 24 hours and some may divide twice. If the incorporation of histone were proportional to the mitotic rate, a difference of 100-fold would be expected between the incorporation into the histone of tumour and liver. The difference found is very much less than this, and from the results of Greenberg and Sassenrath (1955) the distribution of amino acids after intraperitoneal injection appears to be too uniform to introduce a large deviation from the expected difference of this order. Therefore the rate of incorporation into liver histone is greater than could be expected for the synthesis of chromosome histone necessary to maintain the mitotic cycle, and we must conclude that histones undergo an appreciable amount of turnover or additional synthesis.

### SUMMARY

1. The incorporation of *l*-lysine into histones prepared by two different methods and into cytoplasmic proteins of rat and mouse tumours as well as rat and mouse liver has been studied under comparable conditions.

2. The ratio of the specific activity of histone (H) to that of the cytoplasmic proteins (C) of the same cells is greater for the tumours than for the liver of the same animal. However, the differences in these ratios appear to be due to differences of C to an even greater extent than to differences of H and it is possible that in tumour cells the rate of incorporation of amino acids into the cytoplasmic proteins is abnormally low.

3. The differences of rates of incorporation in the tumours and similar normal tissues do not appear to be related to mitotic rates. It is necessary to conclude that a significant turnover or synthesis of histone occurs in resting cells.

We are indebted to Miss P. Simson for carrying out the amino acid analyses given ; to Messrs. B. Mitchley and C. Smith for implanting the tumours.

This investigation has been supported by grants to the Chester Beatty Research Institute (Institute of Cancer Research : Royal Cancer Hospital) from the Medical Research Council, the British Empire Cancer Campaign, the Jane Coffin Childs Memorial Fund for Medical Research, the Anna Fuller Fund, and the National Cancer Institute of the National Institutes of Health, U.S. Public Health Service.

### REFERENCES

BRUNISH, R. AND LUCK, J. M.—(1952) *J. biol. Chem.* **198**, 621.

BUSCH, H., DAVIS, J. R. AND ANDERSON, D. C.—(1958) *Cancer Res.*, **18**, 91[illegible]

*Idem*, DAVIS, J. R., HONIG, G. R., ANDERSON, D. C., NAIR, P. V. AND NYHAN, W. L. —(1959) *Ibid.*, **19**, 1030.

COHN, P. AND BUTLER, J. A. V.—(1958) *Biochem. J.*, **70**, 254.

DALY, M. M., ALLFREY, V. G. AND MIRSKY, A. E.—(1952) *J. gen. Physiol.*, **36**, 173.

DAVIDSON, P. F.—(1957) *Biochem. J.*, **66**, 708.

GREENBERG, D. M. AND SASSENRATH, E. N.—(1955) *Cancer Res.*, **15**, 620.

JOHNS, E. W., PHILLIPS, D. M. P., SIMSON, P. AND BUTLER, J. A. V.—(1960) *Biochem. J.*, **77**, 631.

LOFTFIELD, R. B. AND HARRIS, A.—(1956) *J. biol. Chem.*, **219**, 151.

PHILLIPS, D. M. P. AND JOHNS, E. W.—(1959) *Biochem. J.*, **72**, 538.

ROTHERHAM, J., IRVIN, J. C., IRVIN, E. M. AND HOLBROOK, D. J.—(1957) *Proc. Soc. exp. Biol., N.Y.*, **96**, 21.

SMELLIE, R. M. S., McINDOE, V. M. AND DAVIDSON, J. N.—(1953) *Biochim. biophys. Acta*, **11**, 559.

STEVENS, C. E., DAOUST, R. AND LEBLOND, C. P.—(1953) *J. biol. Chem.*, **202**, 177.